# Stateful Three-Input Logic with Memristive Switches

**DOI:** 10.1038/s41598-019-51039-6

**Published:** 2019-10-10

**Authors:** A. Siemon, R. Drabinski, M. J. Schultis, X. Hu, E. Linn, A. Heittmann, R. Waser, D. Querlioz, S. Menzel, J. S. Friedman

**Affiliations:** 1 Institut für Werkstoffe der Elektrotechnik II (IWE II), RWTH Aachen University, Sommerfeldstr. 24, 52074 Aachen, Germany; 2 JARA-Fundamentals for Future Information Technology, Jülich, Germany; 3 Department of Electrical and Computer Engineering, The University of Texas at Dallas, Richardson, 75080 Texas USA; 4 Peter Grünberg Institut 10 (PGI-10) Forschungszentrum Jülich GmbH, Jülich, Germany; 5 Centre de Nanosciences et de Nanotechnologies, CNRS, Univ. Paris-Sud, Université Paris-Saclay, Palaiseau, 91120 France; 6 Peter Grünberg Institut 7 (PGI-7) Forschungszentrum Jülich GmbH, Jülich, Germany

**Keywords:** Electrical and electronic engineering, Electronic devices

## Abstract

Memristive switches are able to act as both storage and computing elements, which make them an excellent candidate for beyond-CMOS computing. In this paper, multi-input memristive switch logic is proposed, which enables the function X OR (Y NOR Z) to be performed in a single-step with three memristive switches. This ORNOR logic gate increases the capabilities of memristive switches, improving the overall system efficiency of a memristive switch-based computing architecture. Additionally, a computing system architecture and clocking scheme are proposed to further utilize memristive switching for computation. The system architecture is based on a design where multiple computational function blocks are interconnected and controlled by a master clock that synchronizes system data processing and transfer. The clocking steps to perform a full adder with the ORNOR gate are presented along with simulation results using a physics-based model. The full adder function block is integrated into the system architecture to realize a 64-bit full adder, which is also demonstrated through simulation.

## Introduction

The ever-increasing density of transistors in integrated circuits has spurred a revolution of engineering and technology over the last 50 years. With the increase in density quickly approaching its theoretical limits in silicon processes, continued innovation is required to catalyze additional advancements for computing into the next 50 years^1,2^.

With standard CMOS approaching its theoretical limit for minimum feature size, the ability to produce circuits that operate at increasing frequencies is limited due to power dissipation^3^. Furthermore, von Neumann architectures have fundamental speed and power limitations resulting from the continual transfer of data between the processor and memory^4^. This so-called “von Neumann bottleneck” can be avoided if the information required computing an operation is already present within or near the processing unit^5–8^. In particular, if data is stored in the same location in which it is also being processed, a marked increase in calculation speed and decrease in energy dissipation may be achievable^9,10^. The research community is therefore searching for a device and related logic system that can both execute functions and store data in such a non-von Neumann computing architecture.

Memristive switches are particularly promising to aid in the advancement of beyond-CMOS computing^11–13^. Concisely, a memristive device can be switched by appropriate voltage stimuli between at least two different resistance states: a high resistive state (HRS) and a low resistive state (LRS)^14,15^. A very promising class of memristive switches are redox-based memristive switches based on the valence change mechanism (VCM) and the electrochemical metallization mechanism (ECM)^16^. These devices consist of a metal/insulator/metal structure. The motion of charged ions within the insulating layer is the origin of the memristive switching phenomenon. Thus, the switching operation is inherently bipolar. The device switches from the HRS to the LRS (SET operation) with one voltage polarity and back to the HRS (RESET operation) with the other polarity.

Memristive switches have been proposed as building blocks for beyond CMOS computing devices in von Neumann architectures due to their ultrahigh scalability^17–21^. Besides this, they can be exploited for *non*-von Neumann computing architectures. It has been demonstrated that memristive switches are able to compute all standard Boolean logic functions, and therefore are considered functionally complete^17,20,22–28^. Prominent examples are CRS-logic^29,30^, MAGIC logic family^31^ or IMPLY logic^17^. The CRS logic uses as inputs the applied voltages and the resistance and as output the state representation. In contrast, the MAGIC and IMPLY logic families use only the device resistance as inputs and output and are thus stateful logic families.

To advance beyond functional completeness toward a broader logical computation structure, recent work demonstrated the capability of memristive switches to implement adder circuits based on the non-stateful CRS approach^26,27^, and the stateful MAGIC^32^ and IMPLY logic approaches^23,33,34^. Experimentally, an adder based on the CRS logic has been demonstrated using bipolar memristive devices^35^. The functionality of a MAGIC adder has been shown with organic unipolar switching devices^36^. Next to the demonstration of the IMPLY logic in the proposing publication^17^ additional publications presented experimental studies for this approaches^37,38^. All of these adders require a certain number of devices and steps to perform a certain operation, e.g. a 1-bit addition. As memristive switches do not offer an unlimited endurance, reducing the number of steps required for the targeted operation will aid in the advancement of memristive switches as a promising computing technology.

In this paper, we extend the functionality of the IMPLY logic by implementing stateful logic with three devices simultaneously. Three devices are shown to execute the function ${\rm{X}}+\overline{({\rm{Y}}+{\rm{Z}})}$, which will be called the ORNOR gate. We show that this function can aid in reducing the number of steps and improving efficiency in memristive computing. This concept is validated using a physics-based simulation model, which has been fitted to experimental data. This modeling approach enables us to identify possible limitations of the logic approach. The analysis of these limitations paves the way for deducing device and circuit requirements.

## Results

### ORNOR gate

**Figure 1:**
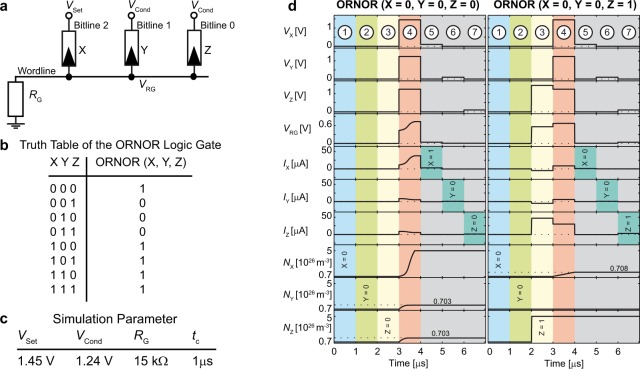
The ORNOR gate’s (**a**) structure and (**b**) truth table. (**c**) Simulation parameter for the circuit of (**a**). (**d**) The simulation of the critical cases of the ORNOR gate are depicted. Here, the subscripts X, Y, Z are indicating the correlation of the applied voltages, currents or state variables to the devices X, Y and Z, respectively. In step 1–3 the initialization process of the ORNOR gate is shown by writing the three inputs to the devices X (blue), Y (light green) and Z (yellow). In step 4 (red) the ORNOR operation is executed, which is followed by three verifying read-out steps (dark green). If a high current is detected the read-out value is a 1, whereas a low current is a 0. Row 1/2/3: voltage applied to the Bitline 2/Bitline 1/Bitline 0. Row 4: potential at the wordline. Row 5/6/7: current at Bitline 2/Bitline 1/Bitline 0. Row 8/9/10: state variable of device X/Y/Z. The scale is changed for small state variable values.

The proposed ORNOR gate can be regarded as an extension of the IMPLY gate proposed by Borghetti *et al*.^17^. In an IMPLY gate, two memristive switches, P and Q are connected via a common node over a resistance to ground. Two different voltages *V*_Set_ and *V*_Cond_ are applied to Q and P, where *V*_Cond_ is not high enough to set P, but *V*_Set_ can set Q in the specific cycle time. By applying these voltages at the same time, the potential at the shared connection is rising depending on the states of the devices. Thus, the voltage drop over Q can be lowered depending on the state of P, so that Q does not switch (see also Supplementary Information). The **ORNOR** gate, in contrast, uses three memristive switches X, Y, and Z as depicted in Fig. 1a. The common line connecting the memristive devices with the resistance *R*_G_ is referred to as wordline in the following. The memristive devices X, Y and Z are contacted via the bitlines 2, 1 and 0, respectively. The conditional voltage *V*_Cond_ is now applied to two devices (Y and Z) and *V*_Set_ is applied to the third device (X). Only the device with *V*_Set_ applied (X) can change states from the HRS to the LRS, but now, two memristive switches (Y and Z) determine the final state of X. The voltage at the *V*_RG_ node is near to *V*_Cond_ relative to ground when either device Y or Z is in the LRS state. In this case, the voltage applied across device X is *V*_Set_ - *V*_Cond_ and therefore does not switch the binary state of X to the LRS. In the case where both Y and Z are in the HRS, the effective voltage at *V*_RG_ is nearly GND, as there is just a tiny current flow through resistor *R*_G_. In this scenario, the voltage across device X is equal to *V*_Set_, which is sufficiently large to change the state of X from the HRS to the LRS. The truth table of this circuit for different inputs (X, Y, and Z) is shown in Fig. 1b. This table can be simplified to the function ${\rm{X}}^{\prime} ={\rm{X}}+\overline{({\rm{Y}}+{\rm{Z}})}$, which will be referred to as the ORNOR gate, as the function is stated as X **OR (**Y **NOR** Z). The output of the function is the state of X after the voltages are applied, written as X’.

To validate the function of the ORNOR gate circuit simulations are performed. For the memristive elements, we used a physics-based simulation model, which is described in detail in the Methods section. The model was fitted to experimental data of a Pt/Ta_2_O_5_/Ta VCM device (see Supplementary Information) and it fulfills the six fundamental criteria required to model VCM devices^39,40^, among which the nonlinear switching kinetics is the most important one. It means that the device will switch upon application of a non-zero voltage in a finite time. It depends, however, on the voltage magnitude how fast the switching will occur. Due to the involved physics, the switching time is a highly nonlinear function of the applied voltage^41^. Since the fastest switching occurs at higher voltages and the target voltages of this application are in the higher voltage range, the fit was chosen to be more accurate in this range.

The simulations of the two critical cases as described below are shown in Fig. 1d. To perform the ORNOR operation, the memristive devices are first initialized to the designated inputs (X blue, Y green and Z yellow), starting in an HRS (steps 1–3). To this end, zero volts are applied to the wordline and the desired inputs to the bitlines. Then, the ORNOR operation (red) is performed in step 4 and afterwards verified by the read-out steps 5–7. The last three rows in Fig. 1d present the state variables of the devices X, Y and Z. Tracking the state variable allows us to observe small state changes, which are hard to detect in the read-out current. Note that the scale is changed for small state variable values.

The critical case 1 (X = 0, Y = 0 and Z = 0) is the only one in which device X switches. Thus, it determines the minimum cycle time *t*_C_. During operation an unwanted state drift occurs. The devices Y and Z, to which *V*_Cond_ is applied, show a small state drift. This state drift is independent of the cycle time since it stops as soon as device X becomes sufficiently low resistive and in turn the potential at *V*_RG_ is high enough. Consequently, the voltage drop over devices Y and Z decreases.

The critical case 2 is X = 0, Y = 0 and Z = 1 and has the same behavior as the case X = 0, Y = 1 and Z = 0. Here, a cycle time dependent state drift of device X is present, since *V*_RG_ is not increased sufficiently by the low ohmic connection of device Z to prevent this drift.

The case X = 0, Y = 1 and Z = 1 is not critical, since the potential *V*_RG_ is even closer to *V*_Cond_ as both devices Y and Z are low ohmic. Thus, the voltage drop over device X decreases and the switching process slows down.

**Figure 2:**
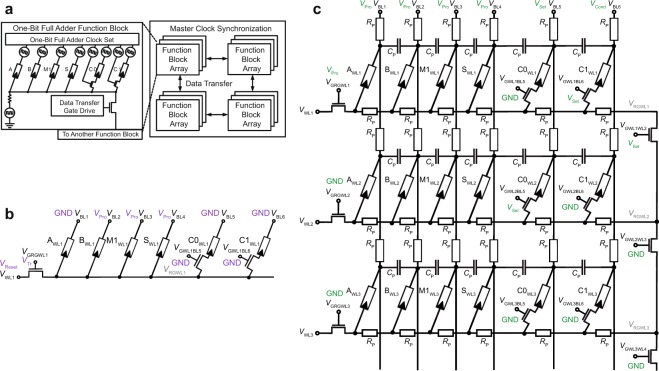
Computing architecture for a memristive computing system. (**a**) The computing system architecture utilizes a synchronized, interconnected array of function blocks to perform complex functions with improved efficiency. Each function block contains a set of memristive switches, which, in conjunction with a set of clock signals, perform a predefined function. For complex computations the data can be processed in multiple function blocks. The transfer between function blocks is done with the data transfer transistor. (**b**) An optimized full adder schematic with six total memristive switches. The signals, which need to be applied for a FALSE operation of device A_WL1_, are displayed in violet. (**c**) Two-Bit adder circuit with parasitic elements. *R*_P_ and *C*_P_ are the parasitic resistors and the parasitic capacitances of the wordlines and bitlines. Here the parasitic capacitances between the wordlines are not depicted due to readability. The signals, which need to be applied for a COPY operation from device C1_WL1_ to C0_WL2_, are displayed in green.

The observed state drift is a direct consequence of the nonlinear switching kinetics of VCM devices, which are included in our device model. Due to a smaller voltage drop over the devices (here in a range of 0.8 V) during the operation the switching process starts in unselected cells^42^. As the switching process is slower in this regime according to the switching kinetics (Fig. 2b), only a small state drift is observed. To alleviate this problem a device with steeper kinetic could be chosen^43^. This effect imposes an additional circuit design constraint on the circuit parameter, i.e. the applied voltages, the timing and the series resistance. The used parameters listed in Fig. 1c were optimized to minimize the drifts and cycle time for performing the ORNOR function.

### Computing system

In the proposed system architecture, arrays of memristive switches are dedicated to performing a specific function. The memristive switches have clock signals applied to their bitlines, as in Fig. 2a. These clocks encode the data for a specific function and drive the memristive switches to compute this function in a serial manner. These arrays are defined as function blocks. An example of one of these function blocks is shown on the left in Fig. 2a, where six memristive switches are set up to compose a full adder. This function block is one of many function blocks that ultimately comprise a computing system based on memristive switches for complex computations. Each of the function blocks repeatedly performs the defined function enabling pipelining^44^. One advantage to this system is that multiple function blocks can be driven by the same set of clocks, thereby performing parallel processing without much overhead in area. Data can be transferred from one function block to another function block for additional computation, or each function block could be reconfigured to perform a different function by changing the clock set. A master clock controls how the various function blocks are synchronized. As shown in Fig. 2a, there is a transistor that gates connections between the common node of one function block and the common node of another function block. Additionally, there are transistors that control the connection of the C0 and C1 memristive switches to this common node, which are specific to the full adder function. The additional transistors enable the transfer of data between function blocks, which use a common clock set for all full adder function blocks.

In this computing system architecture, a full adder circuit is realized. The N-bit full adder circuit proposed in this paper is optimized to exploit the ORNOR function, and requires 6•(N_bit_ + 1) memristive switches. Due to the doubling of the most significant bit to ensure a correct result for a two’s complement addition, an extra full adder functional block (6 devices) is needed. The full adder circuit is realized here with a common transistor at the wordline (see Fig. 2b) instead of a resistor *R*_G_ as in the ORNOR gate (see Fig. 1a). This provides flexibility for using different functions on this block, as the conductance of the transistor can be tuned according to the performed functions (see also Supplementary Information). Figure 2c depicts a 2-bit adder circuit, which is composed of three full adder circuits with common transistor, as an example of a component of the 64-bit adder circuit. The 2-bit adder circuit includes parasitic line resistances *R*_P_ and parasitic capacitances *C*_P_. Here, the parasitic capacitances *C*_p_ between the wordlines are not shown due to readability, but are considered later in the simulations.

To transfer the data from one functional block to another a COPY operation needs to be implemented. During this operation, the data transfer gate drive is set to a conductive state and thus it connects two functional blocks to transfer the data. By performing an IMP operation with two devices, one of each block, the data is transferred to the other block. To copy data of C1_WL1_ to C0_WL2_ in Fig. 2c, the voltage scheme highlighted in color needs to be applied. Since the applied voltages at the bitlines are always applied to all functional blocks, if they are sharing one clock set, selecting transistors must be added to the bitlines that are involved in the COPY operation. Otherwise, *V*_Set_ would be applied to two devices, which share the same bitline and the connected wordlines. In the same way, *V*_Cond_ is applied to two devices sharing another bitline. Thus, no COPY operation would be achieved. By adding the transistors to the circuit, only two devices on the active bitlines can be chosen to be connected to the wordlines by setting the selecting (*V*_GWL1BL6_ and *V*_GWL2BL5_) transistors to a conductive state, here by applying a high voltage *V*_Tr_ to the gates.

For implementing a functionally complete stateful logic system in this architecture, a FALSE operation is required, to switch the memristive devices to 0. When performing the function ORNOR(X, Y, Z) or IMP(P, Q), X and Q are the only memristive switches whose state can be changed. It can be observed from the truth tables that there is no set of inputs that causes the memristive switches X or Q (to which *V*_Set_ have been applied) to change from 1 to 0. Without the FALSE operation, all the memristive switches will eventually be changed to the 1 state, preventing further computation. Since the used memristive switches are bipolar switching devices, a voltage with the opposite polarity of the SET operation needs to be applied to reset the device to the HRS state. To this end, a RESET voltage *V*_Reset_ is applied to the wordline while the bitlines are set either to GND or to *V*_Pro_ in order to reset the device or keep its information. To reset device A in Fig. 2b, the voltage scheme illustrated in purple is applied to the respective terminals.

**Figure 3:**
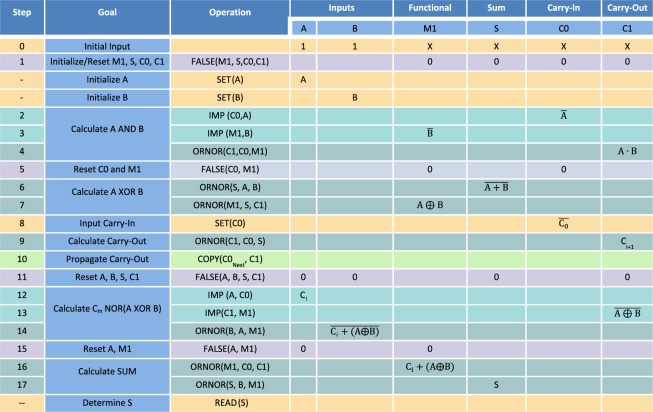
Clocking scheme for a full adder. Each line corresponds to a step in the full adder, with the exception of simulation requirements for loading and transferring data (marked by–). Each step provides the operation that is performed, including the memristive switches that are part of the computation. The result of the operation is shown for each memristive switch, and if there is no value in a cell, it is assumed that the memristive switch maintains its previous state. SET operations are yellow, FALSE operations are violet, COPY operations are light green, IMP operations are green and ORNOR operations are dark green.

The full adder implementation proposed here takes 17 steps to perform a non-K2 one-bit addition, as described in Fig. 3. The step count assumes that setting the initial input values and the final readout are not part of the actual implementation of the function, which is consistent with previous research for a standardized analysis and comparison^23^. These steps are labelled as “–” in Fig. 3 and were required for the simulation. The functions are applied serially to perform the complete full adder function, where data for each step is encoded into the clocks that are applied to the memristive switches. The third column shows the operation of each step, where the memristive switches used in the operations are in parentheses. The outcome of the operation is shown in the column associated with each memristive switch. For example, in step 1, a FALSE operation is applied to the devices M1, S1, C0, and C1. Therefore, the output of each of these memristive switches is shown in their respective columns, where each device state is set to 0.

In general, A, B, and C0 are the memristive switches into which data is loaded, representing the standard A, B, and carry-in for a full adder. Before the execution of the function, data is initially loaded into A and B from another function block using a COPY function and a data transfer transistor. The carry-in is loaded into the C0 memristive switch in step 8. C1 contains the carry-out data of the function block array, and once the computation is complete, S contains the calculated sum. The M1 memristive switch is an additional supporting device. The additional transistors connected to the wordlines of C0 and C1 allow the carry-out of one stage to be shifted to the carry-in of the next stage in a multi-bit chain similar to how a shift register propagates data along a serial chain. Although the memristive switches are defined as inputs or outputs to aid in the explanation of the full adder, every memristive switch can act both as an input or an output based on how they are used.

**Figure 4:**
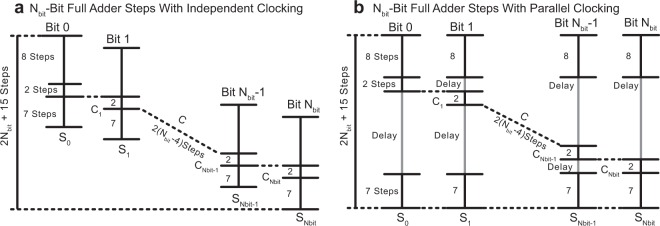
(**a**) Independent clocking and (**b**) parallel clocking scheme. (**a**) In the independent clocking scheme, each functional block has an independent set of clocks, which can be applied at any time to perform the function. In this way, each functional block is aligned so that there is no idle time, however, an increased number of clocks and drivers are required. Approach (**b**) uses the same set of clock signals to drive all memristive switches for the multi-bit addition. The first eight steps and last seven steps are driven across all function blocks simultaneously. This approach is able to compute the multi-bit full addition in the same number of steps while significantly reducing the number of clock signals required due to parallel control of multiple function blocks.

As shown in Fig. 3, with only two steps (9 and 10) of delay between each successive bit of a full-adder, there is increased parallel processing and therefore increased overall efficiency. Two different processing schemes can be realized. The addition is done bit by bit as illustrated in Fig. 4a. In this scheme, the individual bits are processed with an offset of two steps. This scheme, however, requires a unique clock for each memristive switch. In Fig. 4b, the use of the same clock set is coordinated such that the first eight steps and last seven steps are driven between all function blocks simultaneously. This reduces the overall number of drivers required for multi-bit addition.

### Simulations

**Figure 5:**
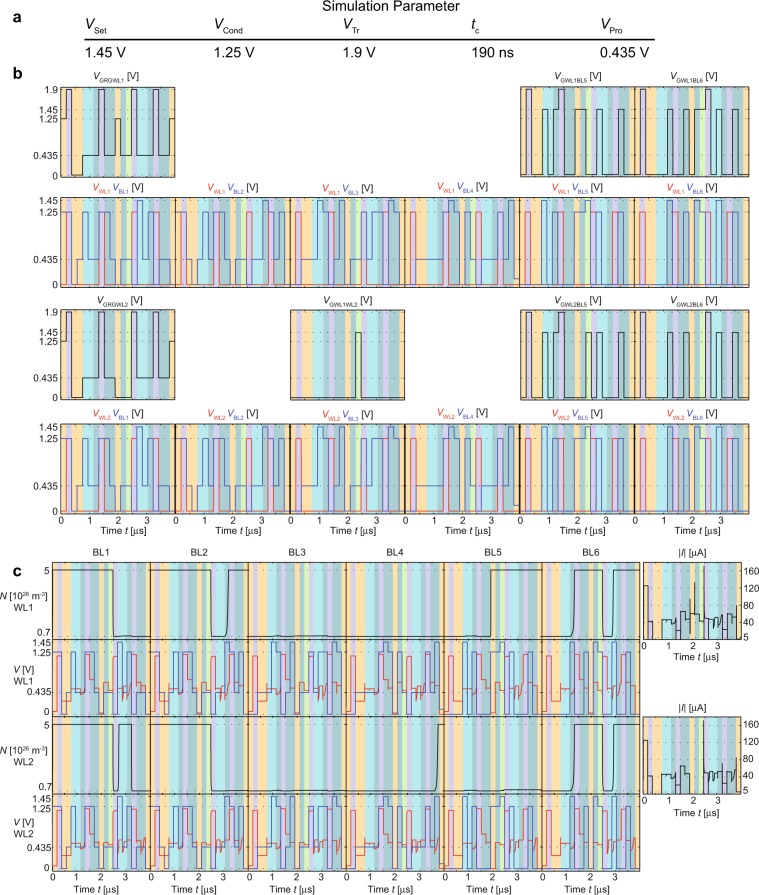
Simulation of a one-bit full adder. (**a**) Circuit parameter. (**b**) Applied voltages. SET processes are yellow, RESET operations are violet COPY operations are light green, IMP operations green and ORNOR operations dark green. Row 1/3: voltage applied to the load transistor and voltages applied to the selecting transistors of WL1/2. Additional in row 3: voltage transient applied to the data transfer gate, which can connect WL1 and WL2. Row 2/4: voltages applied to the bitlines (blue) and the wordlines (red) WL1/WL2. The circuit parameter voltages are marked for better readability. (**c**) State variables and terminal voltages of the one-bit adder. Row 1/3: transient of the state variable and wordline current of WL1/WL2. Row 2/4: wordline potential (red) of WL1/WL2 and voltages applied to the bitline (blue) of each device.

The proposed one-bit adder circuit with parallel clocking scheme is simulated using the model and the model parameters described in the Methods section, the circuit parameters given in Fig. 5a, and the clock scheme introduced in Fig. 3. Using the two’s complement the addition of A = −1 and B = −1 is conducted. To secure a valid result the most significant bit is doubled. The applied voltages are depicted in Fig. 5b. Figure 5c shows the resulting terminal voltages, the change of the state variable of the individual bits during the computation and the currents on the wordlines WL1 and WL2. In the last step of the simulation, the result is read out by applying a small voltage to BL4 (connected to the S devices). For WL1 the detected wordline current is below 1 μA, resulting to a read 0, whereas the detected wordline current for WL2 is above 5 μA and thus interpreted as a 1. This means that the result of (1)_2_  +  (1)_2_ = (10)_2_, which verifies the functionality.

**Figure 6:**
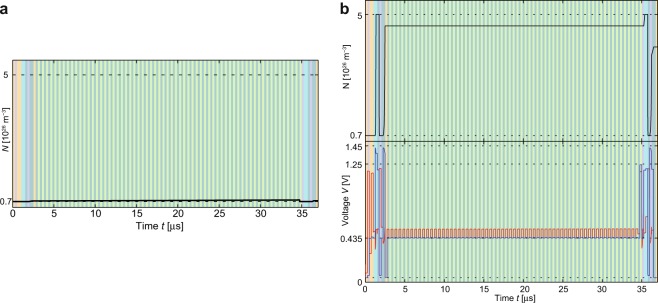
Simulation and comparison of the multibit adder. (**a**) State variable of S_WL65_ under the worst case conditions. The worst case is found to be A – B, where A is 0 and B is 0. The color scheme is adopted from Fig. 3. (**b**) State variable and applied voltage of M1_WL65_ for the worst case simulation. The potential of the wordline is depicted in red and the potential of the connection point at the bitline is shown in blue.

Next, the implementation and verification of a 64-Bit-Adder using the parallel clocking scheme is demonstrated. To ensure proper operation, the worst case in the matter of drift needs to be found first. Since the first eight steps and the last seven steps are executed in parallel, these steps do not differ from the one-bit adder. For multibit operations, however, the steps 9–10 are executed many times. In both steps, the lines BL5 and BL6 are active, but the select transistors only address the required two devices. As the active devices change each repetition in the carry propagation, no drift is expected in these devices. In step 9, BL4 is active, too. The devices connected to BL4 do not have a selector. Thus, a state drift is possible and this effect determines the maximum length of the addition. By means of simulation the worst case is found to be the operation B - A, with A = 0 and B = 0. Figure 6a shows the state variable transition of S_WL65_ for the worst-case operation. Here, a small drift is visible, but the calculated result is still valid. While the result is correct, the state variable of M1_WL65_ is misbehaving as shown in Fig. 6b. Before the carry propagation phase begins, the device M1_WL65_ should have switched to the LRS (*N*_max_) but the state variable does not reach *N*_max_. The reason can be found directly in the applied voltage as the potential on the bitline does not reach *V*_Set_ anymore and thus slows down the switching process. By using a shorter cycle time, the final value of the state will be even lower and eventually the carry would be interpreted as a 0, leading to a malfunction. To ensure proper operation, a cycle time of 250 ns is used here. Extending the cycle time even more to enable a complete switching of M1_WL65_ to *N*_max_ would lead to state drift in other memristive devices.

## Discussion

Previous approaches to calculating a ‘stateful’ full adder focused on solely IMP and FALSE operations, which resets the devices to the HRS^33^, while others^23^ extended this idea by utilizing the XOR operation in both serial and parallel optimized approaches. Table 1 lists the cycle steps and amount of memristive switches as stated in the original papers (if stated). As the adders in the referenced papers use varying methodologies, or count with or without input and output memristive switches, the given quantities have some ambiguity. This ambiguity becomes less important for a large number *N*. In this case, the proposed adder can reduce the needed steps by about 60%. Like in CMOS there is a tradeoff between area (here the amount of memristive switches) and time (here the steps). The amount of memristive switches can be reduced by reducing the parallelism of the algorithm. The number of steps and devices are also a hint towards the power consumption. If the array needs to be bigger (higher number of memristive switches), higher parasitic charging costs and a higher number of sneak paths need to be assumed. Moreover, if more steps are needed to achieve the functionality, more operations with higher voltages are executed on the array and thus the power consumption increases. More details on the energy consumptions are given in the Supplementary Information.

**Table 1:** Comparison of different adder approaches.

Name	Memristive Switches	Steps	Name	Memristive Switches	Steps
Proposed	6 (N_bit_ + 1)	2N_bit_ + 15	MAGIC Conv. Area Optimized^32^	5	15N_bit_
Lehtonen^33^	3N_bit_ + 5	88N_bit_ + 48	MAGIC Conv. Latency Optimized^32^	11N_bit_ − 1	12N_bit_ + 1
Kvatinsky serial^23^	3N_bit_ + 3	29N_bit_	MAGIC Trans. I^32^	22N_bit_ − 3	15N_bit_ + 1
Kvatinsky parallel^23^	9N_bit_	5N_bit_ + 18	MAGIC Trans. II^32^	13N_bit_ − 3	10N_bit_ + 3
Rohani^54^	2N_bit_ + 3	22N_bit_			

Whereas previous work on stateful memristor logic has proposed flexible functionality with enormous overhead control circuit costs^45^, this proposed system architecture employs a parallel clocking scheme that trades functional flexibility for a drastic reduction in the overhead circuit footprint. There is a fundamental relationship between functional flexibility and overhead circuit cost, as functional flexibility requires additional control signals that must be generated by the control circuit. The total transistor count *N*_TR_ of the stateful memristor logic control circuit is given by^45^

$${N}_{TR}=28\,\log \,2(S)+2XS+51X+6S+TS-2,$$

where *S* is the number of steps required performing a particular function, *X* is the number of memristive switches in the circuit, and *T* is the number of select transistors included for functional flexibility. As this overhead circuit cost is quite significant, the proposed system architecture minimizes the overhead circuit footprint by using each control signal to drive a large number of memristive switches and transistors in parallel (Fig. 4b). The decrease in the required number of steps resulting from the use of multi-input memristor logic, in concert with this parallel clocking of function blocks, therefore provide significant improvements to the efficiency of the control circuit and of the system as a whole.

The proposed computing system makes use of the ORNOR gate. The performance improvement relative to using only IMPLY gates is related to the fact that the ORNOR gate is a three input logic gate. The potential of using multi-input gates with three or more memristive devices have been described before^23–25,46^, but the limitations of such circuits could not be addressed partly due to the lack of physics-based simulation models. To allow for multi-input gates, the resistor *R*_G_ needs to be chosen properly. It can be scaled with the number of inputs as proposed in literature^23,46^. In this case, the connection to ground becomes less resistive with each additional input, thus increasingly influencing the switching dynamics of the circuits. If the system is to enable functionality with a wide range in the number of inputs, additional complex periphery circuits must be added due to the scaling of *R*_G_. A second option is to optimize *R*_G_ to enable proper functionality for the two- and three-input gates. Using the simulation model described in the Methods section, we investigated the functionality of multi-input gates for the two different choices of *R*_G_.

**Figure 7:**
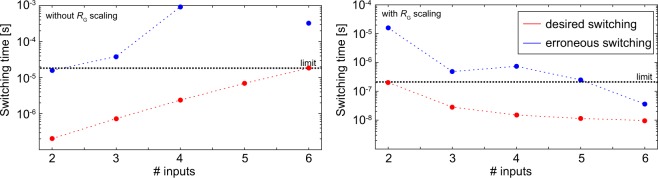
Simulation of the two multi-input approaches with (left) and without (right) *R*_G_ scaling. In both plots, two switching times of the device connected to *V*_Set_ are depicted. In the red case, all devices are in the HRS, and the proper desired functionality is that the device should switch (desired switching). The blue points depicts the switching time if one of the devices connected to *V*_Cond_ is in the LRS. In this case, the device should not switch (erroneous switching). The limit is chosen as the slowest of the desired switching processes; all erroneous switching processes must be slower than this limit.

Figure 7 depicts the simulation results of the slowest desired (red) and fastest erroneous (blue) switching times of gates with various number of inputs. For this study, gates of n-inputs were simulated, where the two-input gate resembles an IMPLY gate and the three-input gate without scaling of *R*_G_ is the proposed ORNOR gate. Thus, an n-input gate includes n memristive switches. Here, n-1 of these switches are connected to *V*_Cond_ whereas always exactly one is connected to *V*_Set_. If *R*_G_ is scaled, n-1 parallel *R*_G_s are assumed in the circuit. If *R*_G_ is not scaled, no additional resistances are added to the circuit. The two worst cases for desired and erroneous switching are simulated for these circuits by applying *V*_Set_ and *V*_Cond_ as constant voltages. Then the switching time of the device connected to *V*_Set_ was analyzed, since it shows the fastest desired and the fastest erroneous switching. The slowest desired switching appears in the case that all devices are in the HRS. The fastest erroneous switching appears if only one device connected to *V*_Cond_ is in the LRS. The limit is set to the slowest desired switching process. All erroneous switching processes must be slower than this limit; hence, all desired switching process need to be completed before an erroneous switching process occurs. Therefore, in both cases the two-input device gates cannot be used in the same circuit with the same voltages and clock period as six-input circuits. This analysis also enables a rough estimation of how many operations can be conducted without additional refreshes. Depending on the minimum time interval between the slowest desired and fastest erroneous switching process, more or fewer sequential steps can operate on the same data without intermediate refresh cycles. This study also represents the stability of this logic approach against variability of the resistance states, since the connection to ground as well as the resistances to *V*_Cond_ are varied over multiple times. As it is depicted in Fig. 7, the scheme is still functioning for reasonable variations.

Figure 7 also depicts the strong influence of *R*_G_, as the desired switching process without scaling of *R*_G_ becomes slower with a higher number of inputs, whereas this process gets faster with increasing number of inputs, if *R*_G_ is scaled. The results presented here are highly dependent on the device characteristic and the circuit parameters. The erroneous switching event is a consequence of the nonlinear switching kinetics of the memristive device. As the device will switch under non-zero voltage input in a finite amount of time, erroneous switching events cannot be avoided completely. Instead, the circuit parameters have to be chosen accordingly. These design constraints can be only deduced when using proper physics-based memristive device models as in this study. In this regard, the circuit parameters must be chosen in concert to ensure that the desired switching process is faster than the erroneous switching process. A desired switching speed can be chosen first, which enables determination of the minimum switching voltage with contemplation of the kinetic characteristic. The set voltage must be higher than this voltage, as there is also a voltage drop over *R*_G_; but this *V*_Set_ must not be too high in order to prevent the device from switching faster in the erroneous switching cases. *V*_Cond_ must also be chosen carefully, as a too high value will cause drift in the devices connected to *V*_Cond_ in the desired switching case, while a too low value causes faster drift of the target device in the erroneous switching case. As can be seen in Fig. 7, a high *R*_G_ value causes a larger voltage drop and slower switching in all cases; a small *R*_G_ value speeds up all switching processes. The optimal circuit parameter can be found by maximizing the time window between the slowest desired and fastest erroneous switching processes.

Moreover, the nonlinear switching dynamics also have to be considered for the cells that do not take part actively in the functional operation. In arrays, a protection voltage *V*_Pro_ that is applied to the unselected devices is required^47^. Depending on the input cases and the states of the unselected devices, *V*_Pro_ has a huge impact on *V*_RG_ and so influences the speed of the operation and the unwanted state drift of the active device. Hence, *V*_Pro_ is also a parameter that needs to be optimized to achieve the best performance.

The 64-Bit-Adder simulation shows, that the desired state of *N*_max_ is not reached (Fig. 6b), but the resulting resistance of the device is nearly unchanged. Hence, the results are still valid. Here, the voltage levels *V*_Set_ and *V*_Cond_ as well as *V*_Pro_ are reduced compared to the optimal values, which are applied by drivers at the one end of the bl near to wl_0_. Since parasitic elements of the wls and bls are included in the simulations, a voltage drop over the lines occurs, resulting in the reduction of applied voltages. Nevertheless, the logic scheme is still functioning, but it may be reasonable to find a better compromise of the circuit parameters for such a setup. Thus, the design is robust against moderate voltage deviations. Next to changing the circuit parameters, the lines could be widened to reduce the line resistance and thus the voltage drop. Moreover, the resistance levels of the memristive devices could be increased, thus less current is flowing over the bl and the line voltage drop is reduced. Due to the included parasitics, also sneak paths and programming disturbances are included in the simulation, but they do not show to have negative effects on the circuit in addition to the voltage drop.

## Conclusions

Memristive switches enable a stateful beyond CMOS computing architecture. A novel extension to current computations with memristive switches is the three-input memristive switch logic gate, named the ORNOR function. A system architecture and clocking scheme have been proposed utilizing the ORNOR function, which enables the memristive switches to perform logic with fewer steps. In particular, a full adder was designed as an element of a multi-bit full adder function; the carry-in-to-carry-out delay was therefore minimized to optimize the overall number of steps required to perform the function. The solution shown here reduces the number of steps by up to 60%, providing a significant improvement in system efficiency. By using a physics-based simulation model, a couple of design constraints could be revealed. The major challenge is to choose the circuit parameters (voltages and cycle times) in a way that enables correct functionality. As memristive devices change their state under non-zero input in a finite time, devices that are not supposed to switch should see small voltage drops only for a limited amount of time. One consequence is that multi-input gates with a large difference in the number of inputs cannot be used with the same clocking scheme. The nonlinearity of the switching kinetics is not identical for all type of memristive devices. Thus, the circuit design parameters will differ when another type of memristive device is used.

## Methods

### Simulation model

Since the physics of VCM devices is still not completely understood, finding an accurate model showing all aspects of memristive switches is an impossible task. There have been initial attempts to characterize the plethora of published ReRAM models and define needed features^39,40^: the most important one being the nonlinear switching kinetics. One model for VCM devices fulfilling these criteria is published by Fleck *et al*.^42^. Here, this model is adapted to model a Pt/TaO_*x*_/Ta device. It is based on the movement of oxygen vacancies within a filamentary region and a concurrent resistance change. The corresponding equivalent circuit model is shown in the Supplementary Information (Fig. 1). In this model, the conductive oxygen-deficient filament is divided into two regions, the disc (light green) and the plug. The plug region is defined as the part of the filament located at the Ta electrode and has a constant high concentration of oxygen vacancies. The disc is located at the Pt electrode and has an oxygen vacancy concentration *N*_disc_ that varies between a minimum concentration *N*_disc,min_ and a maximum concentration *N*_disc,max_. As the resistance is altered by the change of *N*_disc_, this quantity is considered as the state variable. The change of *N*_disc_ is defined as follows:

1 $$\frac{{\rm{d}}{N}_{{\rm{disc}}}}{{\rm{d}}t}=-\,\frac{1}{{z}_{{\rm{Vo}}}eA{l}_{{\rm{disc}}}}{I}_{{\rm{ion}}},$$

where *z*_Vo_ is the charge of the oxygen vacancies, *e* is the elementary charge, *A* is the cross-section of the conducting filament, *l*_disc_ is the length of the disc region, and *I*_ion_ is the ionic current of oxygen vacancies defined at the interface between plug and disc. The ionic conduction can be modeled by a hopping conduction described by the Mott-Gurney law^48^:

2 $${I}_{{\rm{i}}{\rm{o}}{\rm{n}}}=2A{z}_{{\rm{V}}{\rm{o}}}e{c}_{{\rm{V}}{\rm{o}}}a{\nu }_{0}A\,\exp (\frac{-\Delta {W}_{{\rm{A}}}}{{k}_{{\rm{B}}}T})\sinh (\frac{a{z}_{{\rm{V}}{\rm{o}}}e}{2{k}_{{\rm{B}}}T}E)$$

Here, *a* is the hopping distance, *ν*_0_ is the attempt frequency, ∆*W*_A_ is the barrier height for the ion hopping process, *k*_B_ is the Boltzmann constant, *T* is the local temperature, *E* is the electric field, which is considered to be the driving force for the hopping process, and *c*_Vo_ is the mean concentration of plug and disc. This means *c*_Vo_ is modeled by

3 $${c}_{{\rm{Vo}}}=\frac{{N}_{{\rm{plug}}}+{N}_{{\rm{disc}}}}{2}\,$$

with *N*_plug_ being the oxygen vacancy concentration of the plug region. The electric field *E* is given by

4 $$E=\frac{{V}_{{\rm{Schottky}}}+{V}_{{\rm{disc}}}+{V}_{{\rm{plug}}}}{{l}_{{\rm{cell}}}},$$

where *V*_Schottky_ is the voltage drop over the Schottky contact, *V*_disc_ is the voltage drop over the disc region, *V*_plug_ is the voltage drop over the plug, and *l*_cell_ is the oxide layer thickness. For positive voltages, only the thermionic emission is considered as a conduction mechanism of the Schottky contact and is modeled as^49^:

5 $${I}_{{\rm{S}}{\rm{c}}{\rm{h}}{\rm{o}}{\rm{t}}{\rm{t}}{\rm{k}}{\rm{y}},V > 0{\rm{V}}}=A{A}^{\ast }{T}^{2}\,\exp (\frac{-e{\varphi }_{{\rm{B}}{\rm{n}}}}{{k}_{{\rm{B}}}T})(\exp (\frac{e{V}_{{\rm{S}}{\rm{c}}{\rm{h}}{\rm{o}}{\rm{t}}{\rm{t}}{\rm{k}}{\rm{y}}}}{{k}_{{\rm{B}}}T})-1).$$

Here, A* is the Richardson constant and *eϕ*_Bn_ is the effective Schottky barrier height, which is lowered by the image-force lowering effect. The effective Schottky barrier height can be described as follows^49^:

6 $$e{\varphi }_{{\rm{Bn}}}=e{\varphi }_{{\rm{Bn0}}}-e\sqrt[4]{\frac{{e}^{3}{z}_{{\rm{Vo}}}{N}_{{\rm{disc}}}({\varphi }_{{\rm{Bn0}}}-{\varphi }_{{\rm{n}}}-{V}_{{\rm{Schottky}}})}{8{\pi }^{2}{\varepsilon }_{{\varphi }_{B}}^{3}}}$$

with *eϕ*_Bn0_ being the Schottky barrier height under zero bias, *eϕ*_n_ being the difference between the conduction band and the Fermi level, and *ε*_*ϕ*n_ being the effective permittivity in the area of influence of the image-force lowering effect. The Schottky barrier transport mechanism is considered the thermionic-field emission for negative voltages. Thus, the current is calculated by^49^:

7 $${I}_{{\rm{Schottky}},V < {\rm{0}}{\rm{V}}}=-\,A{A}^{\ast }\frac{{T}_{{\rm{S}}}}{{k}_{{\rm{B}}}}\sqrt{\pi {E}_{00}e(-{V}_{{\rm{Schottky}}}+\frac{{\varphi }_{{\rm{Bn}}}}{{\cosh }^{2}(\frac{{E}_{00}}{{k}_{{\rm{B}}}T})})}\,\exp (\frac{-e{\varphi }_{{\rm{Bn}}}}{{E}_{0}})(\exp (\frac{-e{V}_{{\rm{Schottky}}}}{\varepsilon ^{\prime} })-1)$$

with the parameters *E*_00_, *E*_0_ and *ε*′:

8 $${E}_{00}=\frac{eh}{4\pi }\sqrt{\frac{{z}_{{\rm{Vo}}}{N}_{{\rm{disc}}}}{{m}^{\ast }\varepsilon }},$$

9 $${E}_{0}={E}_{00}\,\coth \,(\frac{{E}_{00}}{{k}_{{\rm{B}}}T})$$

and

10 $$\varepsilon ^{\prime} =\frac{{E}_{00}}{({E}_{00}/{k}_{{\rm{B}}}T)-\,\tanh ({E}_{00}/{k}_{{\rm{B}}})}.$$

The contact resistance *R*_contact_ and the plug resistance *R*_plug_ are considered as constant resistances in the model. The contact resistance is supposed to result from the electrodes and the TaO_x_/Ta interface, whereas the plug resistance depends on the geometry of the filament and the assumed oxygen vacancy concentration in the plug region *N*_plug_ and is set to:

11 $${R}_{{\rm{plug}}}=\frac{{l}_{{\rm{plug}}}}{e{z}_{{\rm{Vo}}}{N}_{{\rm{plug}}}{\mu }_{{\rm{n}}}A}$$

where *μ*_n_ is the mobility of the electrons and *l*_plug_ is the length of the plug region. In contrast to the plug and contact resistance, the disc resistance changes with the state variable *N*_disc_ and is calculated as follows:

12 $${R}_{{\rm{disc}}}=\frac{{l}_{{\rm{disc}}}}{e{z}_{{\rm{Vo}}}{N}_{{\rm{disc}}}{\mu }_{{\rm{n}}}A}.$$

Filamentary VCM devices show strong nonlinear kinetics (cmp. Fig. 2b in the supplement). This feature can only be achieved if temperature acceleration is considered^41,50^. Thus, it is important to model the internal temperature:

13 $$T={V}_{{\rm{disc}}}{I}_{{\rm{disc}}}\cdot {R}_{{\rm{th}},{\rm{eff}}}+{T}_{0}$$

where *R*_th,eff_ is the effective thermal resistance of the disc region and *T*_0_ is the ambient temperature.

### Simulation parameters

For this work, the model is fitted to measured kinetic data of a TaO_x_ device for the region of applied voltages (0.5 V–1.3 V) (cmp. Fig. 2b)^51^. For small applied voltages the switching speed of the simulated device differs from the real device about some orders of magnitude. The applied voltages in this paper, however, are inside the fitted region. To estimate the values of the parasitic elements, Cu wires (bitlines/wordline) with a feature size of 40 nm and a height of 40 nm were considered, which are embedded in SiO_2_ as the insulating material. Thus, for a line segment of 80 nm with a spacing of 40 nm and a height of 40 nm the coupling capacitance to the neighboring lines is calculated as *C*_P_ = 2.76•10^−18^ F and the segment resistance is *R*_P_ = 0.86 Ω. The transistors are modeled by a BSIM 4 model with the parameters of^52,53^. The remaining simulation parameters are listed in Table 2.

**Table 2:** Simulation parameter at *T* = *T*_0_.

Symbol		Value	Symbol		Value
*l*_cell_	Insulator layer thickness	5 nm	*R*_contact_	Contact resistance	1 kΩ
*l*_disc_	Length of disc region	3 nm	*R*_th,eff_	Effective thermal resistance	20.2•10^6^ KW^−1^
*A*	Filament area	140 nm^2^	*N*_plug_	Concentration of oxygen vacancies in the plug region	5•10^26^ m^−3^
A*	Richardson constant	11•10^5^ AK^−2^ m^−2^	*N*_disc, max_	Maximum concentration of oxygen vacancies in the disc region	5•10^26^ m^−3^
*ε*	Permittivity	21.5•*ε*_0_	*N*_disc,min_	Minimum concentration of oxygen vacancies in the disc region	0.7•10^26^ m^−3^
*ε*_ϕB_	Permittivity in the Schottky area	11.6•*ε*_0_	*a*	Hopping distance	0.5 nm
*z*_Vo_	Charge of oxygen vacancies	2	*T*_0_	Ambient temperature	293 K
*eϕ*_Bn0_	Schottky barrier height	0.36 eV	*∆W*_A_	Oxygen vacancy activation energy	0.855 eV
*eϕ*_n_	Difference between conduction band and Fermi level	0.1 eV	*μ*_n_	Mobility of electrons	13•10^−6^ m^2^ (Vs)^−1^
*ν*_0_	Attempt frequency	1•10^13^ Hz			

## Supplementary information

Supplementary Info
