# Supplementary material for: Stateful Three-Input Logic with Memristive Switches
Source: Sci Rep. 2019 Oct 10;9:14618. doi: 10.1038/s41598-019-51039-6 (PMC6787102; doi:10.1038/s41598-019-51039-6)
Supplement: Supplementary file 1 — Supplementary Info [file 41598_2019_51039_MOESM1_ESM.docx]

Supplementary Information

to

Stateful Three-Input Logic with Memristive Switches

by

A. Siemon^1,2^, R. Drabinski^1,2^, M. J. Schultis^3^, X. Hu^3^, E. Linn^1,2^, A. Heittmann^4^, R. Waser^1,2,4,6^, D. Querlioz^5^, S. Menzel^2,6*^ and J. S. Friedman^3,5^

^1^ Institut für Werkstoffe der Elektrotechnik II (IWE II), RWTH Aachen University, Sommerfeldstr. 24, 52074 Aachen, Germany

^2^JARA-Fundamentals for Future Information Technology, Jülich, Germany

^3^Department of Electrical and Computer Engineering, The University of Texas at Dallas, Richardson, TX 75080, USA

^4^Peter Grünberg Institut 10 (PGI-10) Forschungszentrum Jülich GmbH, Jülich, Germany

^5^Centre de Nanosciences et de Nanotechnologies, CNRS, Univ. Paris-Sud, Université Paris-Saclay, 91120 Palaiseau, France

^6^Peter Grünberg Institut 7 (PGI-7) Forschungszentrum Jülich GmbH, Jülich, Germany

**Equivalent circuit diagram**

| 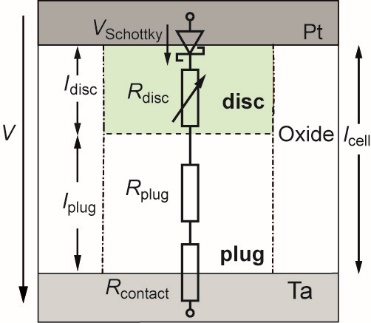 |
| --- |
| FigS. 1: Equivalent circuit diagram of the assumed Pt/TaO_x_/Ta memristive switching device. The conductive filament is divided in a plug and a disc region (light green). |

**Memristive Switches**

Memristive switches can be seen as variable resistances with a simple metal insulator metal (MIM) structure. Normally, they are used in a binary manner, as they switch between a low resistance state (LRS) and a high resistance state (HRS). Since memristive switches are analog devices, they can also be used as multi-state devices ^1-3^.

If a so-called bipolar device is used ^4^, voltages of different polarities need to be applied to the device to switch between the LRS and the HRS as shown in FigS. 2a. In FigS. 2a, a simulated *I-V* characteristic of a Pt/TaO_x_/Ta device is depicted. The device performs a RESET, switching from the LRS to the HRS, when a sufficiently high positive voltage is applied to the Pt electrode; and executes a SET, switching from the HRS to the LRS, for a sufficiently high negative voltage.

| 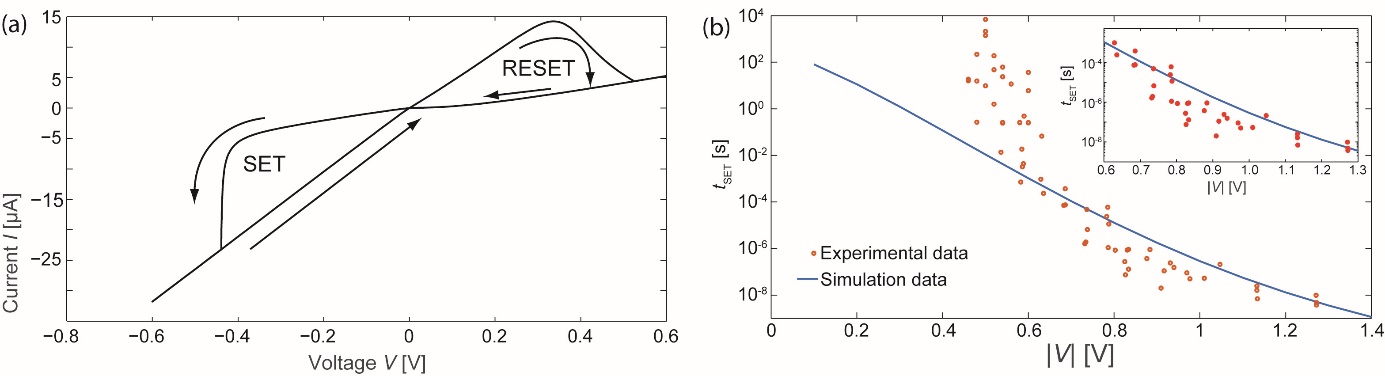 |
| --- |
| FigS. 2 (a) Simulated *I-V* characteristic and (b) switching kinetic of the modeled memristive switch and experimental data taken from ^5^. The voltage is applied to the Pt electrode. |

The two most popular bipolar switching mechanisms for memristive switches are the electrochemical metallization (ECM) and the valance change mechanism (VCM) ^4^. Both mechanisms show filamentary switching. In ECM systems, the filament is grown out of metal ions from the active electrode, whereas in VCM systems the filament region is a region of high oxide sub-stoichiometry, often due to a high concentration of oxygen vacancies. Both systems show nonlinear switching kinetics ^6^ as shown in FigS. 2b, which means that a small change of the applied voltage can result in a difference of some orders of magnitudes of switching time. Since the switching process at small voltages is very slow, sometimes a threshold is assumed ^7-10^. This assumption suggests no change in the resistance state for voltages below the threshold. However, this assumption contradicts the device physics. The limiting electrochemical or physical process defines the switching time ^6^. This process obeys an Arrhenius law, because an energy barrier must be overcome. The barrier lowering depends on the applied electric field. Thus, a finite slope in the log(*t*_SET_)-*V* diagram is expected. If a threshold voltage were present, this slope would be infinite. In fact, even the retention (state stability over time) is a very severe issue ^11-13^. These observations are in clear contrast to the claim of a threshold voltage. Assuming a threshold therefore leads to incorrect predictions of device and circuit behavior, as memristive switches are analog devices with resistance states that can be changed even with a small voltage applied over a sufficient period. Thus, we consider a VCM device with a nonlinear switching kinetic without threshold in this work.

**IMPLY Gate**

In the IMPLY logic approach, the basic operations are the implication function (IMP) and the FALSE operation, which resets the devices to the HRS. The basic circuit for these functions is shown in FigS. 3. It consists of two memristive switches P, Q, and one resistor *R*_G_. In the circuit, both memristive devices are connected to the same wordline, whereas the bitlines are different for each device. The states of the memristive switches are interpreted as the logic inputs and output of the function, where the LRS is equal to a logical one 1 and the HRS to a logical zero 0. Since all logic inputs are stored in the device state, and do not need to be read out, this logic is considered a ‘stateful’ logic.

The memristive switches are assumed ideal threshold switches with the thresholds *V*_CLOSE_ and *V*_OPEN_ ^14^. The proposed circuit is constructed to meet the device assumption that a sufficiently large negative voltage (|*V*| > |*V*_CLOSE_|) applied to the bitlines sets the device, and a sufficiently large positive voltage (*V* > *V*_OPEN_) resets the device.

The IMP function can be implemented by applying a conditional voltage *V*_Cond_ that is higher than *V*_CLOSE_ to device P, and a set voltage *V*_Set_ that is lower than *V*_CLOSE_ to device Q (|*V*_Cond_| < |*V*_CLOSE_| < |*V*_Set_|). The result is stored in Q after the operation. Since the applied voltages can only set Q, there are only two interesting input combinations, for which Q is 0. If P and Q are both 0, nearly the whole voltage *V*_Cond_ drops over P and the potential *V*_RG_ is close to 0 V. Consequently, almost the whole voltage *V*_Set_ drops over Q, which causes Q to switch to 1. If P is 1 and Q is 0, nearly the whole voltage drops over the resistance *R*_G_ and the potential *V*_RG_ rises to a value close to *V*_Cond_. In this case, the voltage across Q is higher than *V*_CLOSE_ and Q remains 0. Following this explanation with simplified device behavior already one constraint is visible: *R*_LRS_ << *R*_G_ << *R*_HRS_.

The used device assumptions in ^14^ lead to two discrepancies compared to real memristive switches:

### Real devices are analog, not only two resistance states exist.

### Real devices do not offer a threshold.

Realistic devices must consider additional constraints, especially if the operation is conducted in arrays with more devices than needed for the gate ^15, 16^.

| 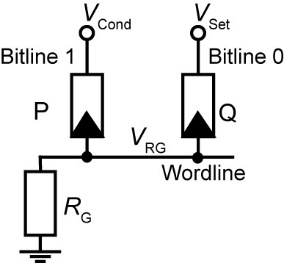 |
| --- |
| FigS. 3 Circuit of an IMP gate using the IMPLY logic approach. |

In FigS. 4 the simulation results of the two interesting cases of the IMP function are depicted using the introduced model and the circuit parameters of Table S1. In the first two steps, the devices P (blue) and Q (green) are initialized by adjusting the state corresponding to the inputs. In this simulation, the devices are in the HRS at the start of the simulation. By applying a 0 to the wordline and the desired inputs to the bitlines, the states are adjusted. In the third step, the IMP operation (red) is conducted and in the last two steps, the states of P and Q are read out to verify the functionality. Here a high current is defined as 1 and a low current as 0. The first two rows in FigS. 4 depict the input signals applied to the individual bitlines. In the third row, the potential of the wordline is shown and the fourth and fifth row present the corresponding bitline currents. In row six and seven, the state variables of the two devices are displayed. Here also small changes, which are not visible in the current answer, can be seen. Note that the scale is magnified for small state variable values for better visualization.

Table SI Circuit design parameter

| *V*_Set_ | *V*_Cond_ | *R*_G_ | *t*_c_ |
| --- | --- | --- | --- |
| 1.45 V | 1.24 V | 15 kΩ | 250 ns |

Since the (P = 0; Q = 0)-case is the only case in which a change of device states should appear, it determines the cycle timing *t*_c_. Depending on the chosen timing and voltages, unwanted ‘state drifts’ can appear ^16^. In case P = 0 and Q = 0, the red box highlights the drift in P and in case P = 1, Q = 0 the drift in Q is highlighted. These drifts appear due to the threshold-free nature of the devices and show the need of refresh cycles, good timing, and precise voltage control for this kind of logic. These drifts and the circuit parameter of Table SI are heavily dependent on the used device or assumed device characteristic and therefore are optimized. In contrast to the drift in the case P = 1, Q = 0, the drift in the case P = 0, Q = 0 is mostly independent of the chosen timing, since the drift process stops when Q is sufficiently low ohmic. Due to the switching of Q, the potential *V*_RG_ increases and the voltage drop over P decreases. Thus, the switching process slows down.

To use this logic approach in arrays, the authors of ^15^showed the need of a protection voltage *V*_Pro_, which is applied to the unselected devices. Depending on the input cases and the states of the unselected devices, *V*_Pro_ has a huge impact on *V*_RG_ and so influences the speed of the operation and the unwanted state drift. Hence, *V*_Pro_ is also a parameter that needs to be optimized to achieve the best performance.

| 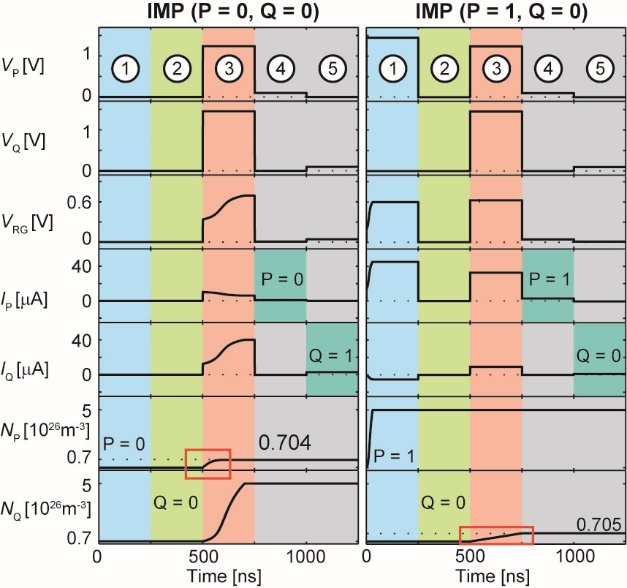 |
| --- |
| FigS. 4 Critical cases for a Two-Input-IMPLY logic gate. In step 1 and 2 the inputs are written to the devices (blue and light green). In the third step the IMP operation is performed. Step 4 and 5 verify the right results by performing a read-out of the two devices (dark green). If a high current is detected a 1 is read, whereas a low current is interpreted as a 0. Row 1/2: voltage applied to Bitline 1/Bitline 0. Row 3: potential at the wordline. Row 4/5: current at Bitline 0/Bitline 1. Row 6/7: state variable of device P/Q. The scale is magnified for small state variable values for better visualization. The red boxes highlight the state drift of the two devices, which occurs during the operation. |

**COPY operation with load resistor**

In principle, the COPY operation in the computing system can be also performed with the full adder circuit using a common resistor to GND. In this case, however, the overall resistance to GND is *R*_G_/2 as the two wordlines are connected via the pass transistor. This leads to different voltage drops over the memristive devices as when performing an operation only on one functional block, e.g. ORNOR or IMP. Thus, the circuit parameters, which have been optimized for the full adder circuit, are suboptimal for the connection of two blocks and could lead to strong unintended state drift. Using a common transistor to GND offers the possibility to adjust the resistance to GND according to the executed function. During the COPY operation, one of the two transistor is blocking and its connection to GND is resistively decoupled.

**FALSE operation with load resistor**

Using the common transistor in this circuit is advantageous over the use of a common resistor *R*_G_ as the resistance to GND can be tuned. In contrast, to the ORNOR or IMP function a very low ohmic connection to the wordline terminal is favored to allow for a low *V*_Reset_. Since *R*_G_ was optimized for the ORNOR operation to a value close to the LRS, nearly half the voltage would drops over *R*_G_ and *V*_Reset_ needs to be about twice as high. If *V*_Reset_ is doubled, the potential *V*_RG_ does not double. The potential *V*_RG_ is strongly influenced by *V*_Pro_, especially, if the devices connected to *V*_Pro_ are in the LRS. For this circuit it would be thus preferable to have a higher *V*_Pro_ than for the other operations. Using the common transistor avoids these problems. For the ORNOR and the IMP operation, *V*_Pro_ is applied to its gate. Since the potential *V*_RG_ only varies a little bit, the resistance of this configuration is nearly stable. Applying a very high voltage *V*_TR_ to the common wordline transistor gate sets the transistor to a very good conducting state. Thus, the voltage divider effect can be neglected.

**Energy consumption**

The energy consumption *E*_total_ consists of the energy of the array *E*_array_ and the energy of the periphery *E*_periphery_ (*E*_total_ = *E*_array_ + *E*_periphery_). Our proposed one-bit adder example has an energy consumption of *E*_array_ = 434.7 pJ, which is conceived from the circuit simulation. The given value cannot be easily used for comparison. For a valid comparison, the different adder schemes need to be simulated using the same ReRAM model and the same circuit parameters. In addition, the different schemes may require different peripheral circuitry, which need to be evaluated as well for a reasonable comparison.

A more abstract view on the energy consumption of the array could be reached by giving an energy estimation by measuring the mean energy of the single instructions and multiply it with the number of occurrences in the adder scheme. Here, a full adder consists of two FALSE operation with four devices, two FALSE operation with two devices, one copy operation, one SET operation, four IMPLY operations and seven ORNOR operations. The problem with this figure of merit is that the energy consumption does not only depend on the involved devices, but also on the leakage through the other devices. Thus, the energy consumption will change with the array size and cannot be obtained considering the single instructions only. Therefore, the energy consumption of different adder schemes cannot be easily estimated and compared.

1. Chien, W.-C. *et al.* A Multi-Level 40nm WO_x_ Resistive Memory with Excellent Reliability. *2011 IEEE International Electron Devices Meeting (IEDM), Washington, DC, USA, 5-7 Dec. 2011* (2011).

2. Chien, W. C. *et al.* Multi-Level Operation of Fully CMOS Compatible WOX Resistive Random Access Memory (RRAM). *2009 IEEE International Memory Workshop, Monterey, CA, 10-14 May 2009*, 15-16 (2009).

3. Kim, W., Chattopadhyay, A., Siemon, A., Linn, E., Waser, R. & Rana, V. Multistate Memristive Tantalum Oxide Devices for Ternary Arithmetic. *Sci. Rep.* **6**, 36652 (2016).

4. Waser, R., Dittmann, R., Staikov, G. & Szot, K. Redox-Based Resistive Switching Memories - Nanoionic Mechanisms, Prospects, and Challenges. *Adv. Mater.* **21**, 2632-2663 (2009).

5. Havel, V. *et al.* Ultrafast Switching in Ta_2_O_5_-based Resistive Memories. *Silicon Nanoelectronics Worshop SNW 2016, Hawaii*, 82-83 (2016).

6. Menzel, S., Salinga, M., Böttger, U. & Wimmer, M. Physics of the switching kinetics in resistive memories. *Adv. Funct. Mater.* **25**, 6306-6325 (2015).

7. Kvatinsky, S., Ramadan, M., Friedman, E. G. & Kolodny, A. VTEAM: A General Model for Voltage-Controlled Memristors. *IEEE Trans. Circuits Syst. II-Express Briefs* **68**, 786-790 (2015).

8. Kvatinsky, S., Friedman, E. G., Kolodny, A. & Weiser, U. C. TEAM: ThrEshold Adaptive Memristor Model. *IEEE Trans. Circuits Syst. I-Regul. Pap.* **60**, 211-221 (2013).

9. Yakopcic, C., Taha, T. M., Subramanyam, G. & Pino, R. E. Generalized Memristive Device SPICE Model and its Application in Circuit Design. *IEEE Transactions on Computer-Aided Design of Integrated Circuits and Systems* **32**, 1201-1214 (2013).

10. Pino, R. E. *et al.* Compact method for modeling and simulation of memristor devices: Ion conductor chalcogenide-based memristor devices. *2010 IEEE/ACM International Symposium on Nanoscale Architectures, Anaheim, CA, USA, June 17 - 18, 2010*, 1-4 (2010).

11. Ninomiya, T., Wei, Z., Muraoka, S., Yasuhara, R., Katayama, K. & Takagi, T. Conductive Filament Scaling of TaOx Bipolar ReRAM for Improving Data Retention Under Low Operation Current. *IEEE Trans. Electron Devices* **60**, 1384-1389 (2013).

12. Ninomiya, T., Muraoka, S., Wei, Z., Yasuhara, R., Katayama, K. & Takagi, T. Improvement of Data Retention During Long-Term Use by Suppressing Conductive Filament Expansion in TaOx Bipolar-ReRAM. *IEEE Electron Device Lett.* **34**, 762-764 (2013).

13. Chen, Y. *et al.* Endurance/retention trade-off on HfO2\metal cap 1T1R bipolar RRAM. *IEEE Trans. Electron Devices* **60**, 1114-1121 (2013).

14. Borghetti, J., Snider, G. S., Kuekes, P. J., Yang, J. J., Stewart, D. R. & Williams, R. S. ‘Memristive’ switches enable ‘stateful’ logic operations via material implication. *Nature* **464**, 873-876 (2010).

15. Zhu, X., Yang, X., Wu, C., Xiao, N., Wu, J. & Yi, X. Performing Stateful Logic on Memristor Memory. *IEEE Transactions on Circuits and Systems Part II – Express Briefs* **60**, 682-686 (2013).

16. Kvatinsky, S., Friedman, E. G., Kolodny, A. & Weiser, U. C. Memristor-Based Material Implication (IMPLY) Logic: Design Principles and Methodologies. *IEEE Transactions on Very Large Scale Integration (VLSI) Systems* **22**, 2054-2066 (2014).
